# Comparison of short-term clinical results between modified kinematically-aligned and guided motion bicruciate stabilized total knee arthroplasty

**DOI:** 10.1186/s42836-024-00257-z

**Published:** 2024-07-04

**Authors:** Kensuke Anjiki, Naoki Nakano, Kazunari Ishida, Koji Takayama, Masahiro Fujita, Tomoyuki Kamenaga, Masanori Tsubosaka, Yuichi Kuroda, Shinya Hayashi, Ryosuke Kuroda, Tomoyuki Matsumoto

**Affiliations:** 1 Department of Orthopedic Surgery, Kobe University Graduate School of Medicine, Kobe, 650-0017 Japan; 2 Department of Orthopaedic Surgery, Kobe Kaisei Hospital, Kobe, 657-0068 Japan; 3 Department of Orthopaedic Surgery, Takayama Orthopedic Clinic, Kobe, 654-0049 Japan

**Keywords:** Total knee arthroplasty, Kinematically-aligned, Guided-motion, Rotational mismatch

## Abstract

**Background:**

Both kinematically-aligned (KA) total knee arthroplasty (TKA) and bicruciate stabilized (BCS) TKA aim to reproduce the physiological knee kinematics. In this study, we compared the femoro-tibial component rotational mismatch between patients who underwent modified KA-TKA and those who received guided-motion BCS-TKA, and its influence on the clinical outcomes.

**Methods:**

In this retrospective study, 77 consecutive patients were included and divided into two groups: subjects who underwent modified KA-TKA with Persona (KA Group; *n* = 42) and those who received BCS-TKA with JOURNEY II (BCS group; *n* = 35). Range of motion, the 2011 Knee Society Score (KSS), the rotational alignment of the femoral and tibial components, and the correlations between the rotational mismatch and the 2011 KSS subscales were examined.

**Results:**

The postoperative objective knee indicators (*P* = 0.0157), patient satisfaction (*P* = 0.0039) and functional activity scores (*P* = 0.0013) in the KA group were significantly superior to those in the BCS group 1 year postoperatively. There was no significant difference between the two groups observed in the rotational mismatch. In the BCS group, significant negative correlations were identified between the rotational mismatch and objective indicators, patient satisfaction, and functional activity scores but not in the KA group.

**Conclusions:**

The short-term clinical results following KA-TKA showed superior objective knee indicators, patient satisfaction and functional activity scores. A negative correlation was observed between component rotational mismatch and the 2011 KSS subscales in the BCS group, compared to no relationship found between the two in the KA group. These findings suggested that KA-TKA has a relatively higher tolerance for rotational mismatch than BCS-TKA.

## Introduction

Total knee arthroplasty (TKA) demonstrates both safety and efficiency as a surgical procedure for treating advanced knee arthritis [1], and primary TKA can achieve long-term implant survival [2]. However, a high survival rate does not always correlate with superior patient satisfaction or functional outcomes [3, 4], with some studies indicating that about 20% of patients who undergo TKA are dissatisfied afterward [4, 5]. Dissatisfaction and inferior functional results are attributed to some anomalous kinematics of the modern prosthetic design, compared to natural knee kinematics [6, 7].

Kinematically-aligned TKA (KA-TKA) has garnered increased interest for potentially outperforming mechanically aligned TKA (MA-TKA) [8–10]. KA-TKA aims to mirror the natural lower limb alignment and joint surface orientation before arthritis by resurfacing the joint via osteotomy to be of the same thickness as the implant, with only exceptional soft tissue release [9]. Further, the femoral and tibial components are placed in a way that restores the angles and levels of the distal and posterior femoral and tibial joint lines to their respective pre-arthritic condition to avoid neutral limb alignment, which is unnatural to most patients [11, 12]. KA-TKAs are reported to reproduce the physiological knee kinematics, such as medial pivoting and bilateral rollback of the femur [13–15]. In a recent report comparing KA-TKA and MA-TKA, surgical assessment of soft tissue balance and kinematics confirmed medial stability in the entire range of motion (ROM) while maintaining lateral laxity and tibial internal rotation in the flexion region, resulting in good flexion angle and clinical results with KA-TKA [16–18].

Conversely, a surgical procedure aiming to reproduce the physiological knee kinematics using a different approach is an operation that employs the JOURNEY II BiCruciate Stabilized (BCS) Total Knee System, manufactured by Smith & Nephew (Memphis, TN, USA). This knee system consists of an asymmetrical femoral component, a 3° tibial varus angle-matching polyethylene insert, and medial concave shape with a slightly convex (lateral) contour to reproduce normal knee kinematics through mechanically constrained guided motion, including medial pivoting and bilateral rollback of the femur. Post-cam mechanisms mimic the roles of the anterior and posterior cruciate ligaments (ACL and PCL). In addition, the cam and post are asymmetric, directing tibial external rotation concerning the femur during flexion [19, 20]. The JOURNEY II BCS is an enhanced second generation guided-motion total knee system [21, 22], demonstrating favorable short- and medium-term outcomes [23, 24]. Additionally, knee kinematics in BCS-TKAs was excellently reproduced 2 years postoperatively when compared with cruciate-retaining TKAs also performed using the JOURNEY II [25].

The primary goals of the KA- and BCS-TKAs have been to reproduce more physiological knee kinematics. The concept of rotational alignment varies with the two surgical procedures due to their difference in rotational placement of the femoral component. Precise component alignment in TKA is vital, including the tibial and femoral component rotational alignments. A rotational mismatch between these components poses a considerable risk that can cause impaired function and post-TKA dissatisfaction that results from pain, stiffness, polyethylene wear, and patellofemoral joint complications [26, 27].

Therefore, in this study, we focused on the rotational mismatch and compared its influence on clinical outcomes in patients who received modified KA-TKAs and those who underwent guided-motion BCS-TKAs. We hypothesized that the tolerance for rotational mismatch differs in patients who undergo modified KA-TKA and those who received guided-motion BCS-TKA, which affects their clinical outcomes.

## Methods

### Study design

This retrospective cohort study obtained approval from the institutional review board. The inclusion criteria were as follows: severe pain and a decline in function due to grades 3 and 4 knee osteoarthritis (OA) as classified by Kellgren-Lawrence, or subsequent failed conservative treatments. We excluded those with knees exhibiting valgus deformities, severe varus deformity > 20°, PCL dysfunction, extensive bony defects that demanded bone grafting or augmentation, revision TKAs, ongoing knee joint infection, or those receiving bilateral TKAs. Between September 2017 and February 2019, a total of 77 consecutive patients (68 women and 9 men) were enrolled in the study. Patients were divided into two groups: 42 underwent modified KA-TKA with Persona (Zimmer Biomet Inc., Warsaw, IN, USA; KA group) between September 2017 and May 2018, and 35 patients underwent BCS-TKA with JOURNEY II (Smith and Nephew Inc, Memphis, TN, USA; BCS group) between May 2018 and February 2019. One senior surgeon performed all surgeries. There were no differences in the patient characteristics between the groups (Table 1).

**Table 1:** Patient characteristics

**Characteristics**	**KA**	**BCS**	***P*-value**
**Number of cases**	42	35	
**Sex, female/male**	37/5	31/4	N.S
**Diagnosis, (%OA)**	100%	100%	N.S
**Age, years**	73.4 ± 7.9	75.9 ± 7.4	N.S
**Height, cm**	151.8 ± 7.6	151.2 ± 4.8	N.S
**Weight, kg**	62.5 ± 9.7	61.1 ± 8.9	N.S
**Body mass index, kg/m**^**2**^	27.0 ± 3.1	26.7 ± 3.8	N.S
**Deformity (Varus), °**^**a**^	9.6 ± 4.7	9.3 ± 5.2	N.S
**Range of motion**			
**Extension, °**	−12.6 ± 5.6	−10.4 ± 6.8	N.S
**Flexion, °**	116.0 ± 14.1	122.3 ± 13.0	N.S
**2011 Knee Society Score**			
**Objective knee indicators (100)**	57.7 ± 16.9	62.1 ± 17.1	N.S
**Patient satisfaction (40)**	13.9 ± 7.7	15.3 ± 5.0	N.S
**Patient expectations (15)**	12.2 ± 2.5	11.6 ± 2.5	N.S
**Functional activities (100)**	41.9 ± 16.0	42.4 ± 14.8	N.S

The data are given as mean ± standard deviation

*KA* Knee arthroplasty, *BCS* Bicruciate stabilized, *OA* Osteoarthritis, *N.S.* Not Significant

^a^Positive values indicate varus alignment

### Surgical procedures

For both KA-TKA and BCS-TKA, all surgeries began after tourniquet inflation. The knee joint was accessed via a medial parapatellar approach, and the patella remained un-everted.

In KA-TKA, the ACL was removed, while conserving PCL insertion. PCL functionality was confirmed preoperatively on epicondylar-view radiographs and computed tomography (CT) images of the intercondylar osteophytes. Intraoperatively, PCL insertion was reaffirmed and conserved using a bony island. Patella modifications included trimming and reshaping for better alignment with the femoral component’s trochlea by removing a section of the patella’s lateral surface and encircling osteophytes. For the tibia-restricted modification of KA-TKA using iASSIST (Zimmer Biomet, Warsaw, IN, USA) accelerometer-based portable navigation, a 3° varus and a 7° posterior tilt osteotomy of tibia were conducted relative to the mechanical axis. This technique differs from that of the original KA-TKA procedure performed with a generic instrument [9]. A 3° varus of the coronal plane was selected, considering observed tibial plateau tilts in asymptomatic volunteers across different age groups, to avoid extensive varus tibial component transplantation. On sagittal plane, a 7° posterior slope was determined, aligning with reported posterior slopes in normal knees [28, 29]. Subsequently, a femoral osteotomy was performed using a conventional osteotomy guide, with the cartilage wear taken into consideration during operation. For the posterior condyle osteotomy, an appropriate component size was selected mirroring the femoral component’s condyle (medial/lateral 9 mm) and being aligned parallel to the posterior condyle axis. The tibial rotational axis was determined using the ROM technique [30]. This involved moving the knee over the full ranges of flexion and extension with the tibial tray in a floating position. Afterwards, the proper position of the tibial component trial relative to the femoral component was confirmed, and the tibial rotation was determined by making a final adjustment using the Akagi’s line from the medial edge of the tibial tubercle to the midpoint of the PCL [31].

BCS-TKA replicates kinematics via guided-motion aligned with the anatomically-compatible femoral component and inserts configuration. Thus, an osteotomy was performed following the MA-TKA principles. The surgeon resected the ACL and PCL, and a distal femoral osteotomy was conducted perpendicular to the mechanical axis of the femur. Next, a proximal tibial osteotomy was done, involving a 10-mm bone resection on the lateral tibial plateau and a 5° posterior slope on the sagittal plane, perpendicular to the mechanical axis on the coronal plane. Posterior femoral resection, with a 3° external rotation relative to the posterior condylar axis, was carried out utilizing a conventional guide after verifying neutral alignment with each cut in the distal femur and proximal tibia. Tibial rotation was determined by referencing Akagi’s line.

### Clinical evaluation

Each patient was clinically evaluated 1 year postoperatively. The 2011 KSS includes a score from the surgeon and a score from the patient. The surgeon’s score relates to the objective knee indicator score in the 2011 KSS, encompassing factors such as alignment, instability, and joint motion. The patient’s score involves four subscales in the 2011 KSS: objective knee indicator, patient satisfaction, patient expectations, and functional activities. Additionally, the study compared surgical time, intraoperative blood loss, anesthesia time, amount of anesthesics used, postoperative pain management and use of analgesics, surgical equipment costs, rehabilitation costs, and patient medical costs between the two groups.

### Postoperative measurement of the component alignments

**Fig. 1:**
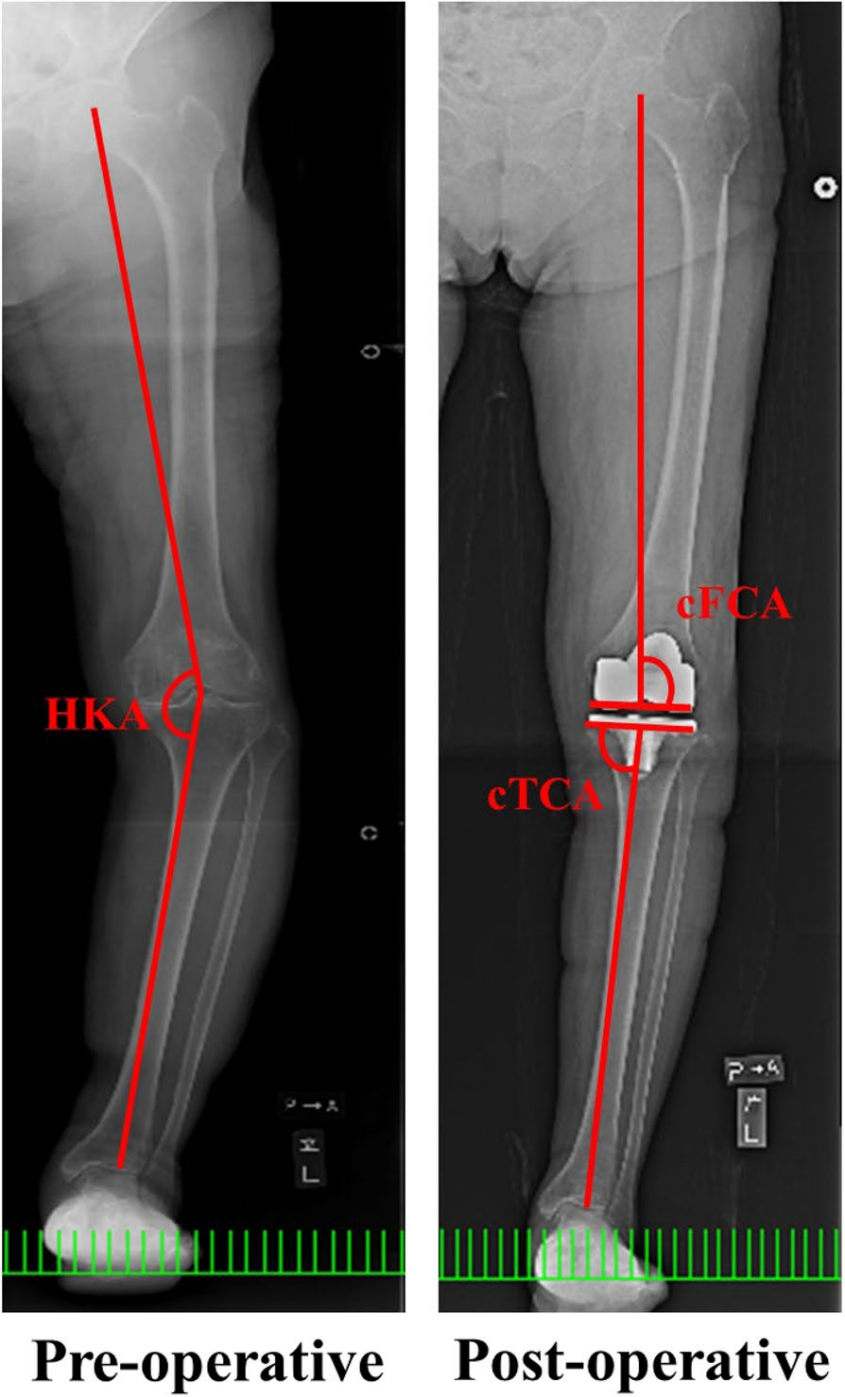
Pre- and postoperative X-rays of a patient who underwent restricted KA-TKA along with a radiographic evaluation. Hip‐knee‐ankle (HKA) angle is the internal angle formed by the femur and tibia’s mechanical lines. The coronal femoral component angle (cFCA) is defined as the external angle formed by the femoral mechanical axis and the tangent to the most distal part of the medial and lateral condyles of the femoral component on the coronal plane. The coronal tibial component angle (cTCA) is defined as the internal angle formed by the tibial mechanical axis and the tangent to the plateau of the tibial component on the coronal plane

One month postoperatively, a full-length double-leg standing posteroanterior radiography was performed. Postoperative measurements included the hip-knee-ankle (HKA) angle, coronal femoral component angle (cFCA), and coronal tibial component angle (cTCA) (Fig. 1). Postoperative CT scans were conducted from the pelvis to the ankle joint and subsequently transferred to a three-dimensional template software package (Zed Knee; Lexi, Tokyo, Japan). Implant models in the computer-aided design were manually adjusted for postoperative multiplanar reconstruction on CT images.

**Fig. 2:**
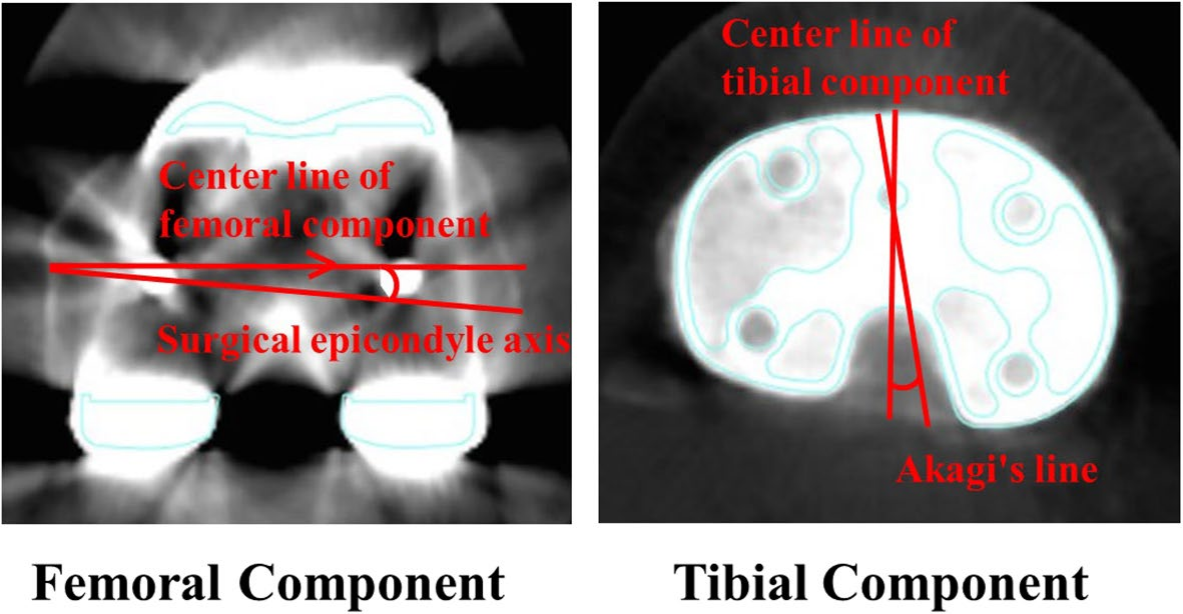
Femoral and tibial component rotational angle. Axial computed tomography (CT) image of the femur. The surgical epicondylar axis (SEA) connects the lowest point of the medial epicondyle to the midpoint of the lateral epicondyle. The prosthetic posterior condylar line (PCL) connects the medial and lateral prosthetic posterior condylar surfaces. The femoral component rotational angle was defined as the angle between the SEA and the PCL. Axial CT image of the tibia. Akagi’s line connects the center of the posterior cruciate ligament and the medial border of the tibial tuberosity. The tibial component rotational angle was defined as the angle between the centerline of the tibial component and Akagi’s line. The difference in rotational angles between the femoral and tibial components constituted the rotational mismatch

The femoral rotational index was determined as the surgical epicondylar axis (SEA), identified by the line connecting the ridge of the lateral epicondyle to the lowest point of the medial groove in the medial epicondyle [32]. The antero-posterior (AP) axis of the femur was a line passing through the midpoint and perpendicular to the surgical epicondylar axis (SEA). The tibial component’s rotational orientation referred to Akagi’s line [31] as AP axis, defined as the line connecting the PCL midpoint attachment to the medial boundary of the patella tendon in the tibial attachment (Fig. 2). The difference in rotational angles between the femoral and tibial components constituted the rotational mismatch [33]. A positive value indicated an external rotational position, while a negative value denoted an internal rotational position.

### Statistical analyses

Two investigators (KA and MF) conducted radiological and CT assessments twice to evaluate intra- and inter-observer reliability for radiographic measurements. All measurements showed a reliability of > 0.85 using intra-class correlation coefficients (ranging from 0.86 to 0.97). The statistical analysis was conducted using a statistical software package (EZR, Saitama Medical Center, Jichi Medical University, Saitama, Japan) [34]. Knee extension-flexion angles, KSS subscales, and parameters of X-ray and CT measurements exhibited normal distribution. Parameters between the two groups were compared using the unpaired *t*-test for numerical values, while Fisher’s exact test was used for qualitative variables. The correlation between the rotational mismatch and the 2011 KSS in both groups was investigated using Pearson’s correlation coefficient to explore the influence of a component rotational mismatch on clinical outcomes. *P* < 0.05 was considered statistically significant. Posthoc power analysis was conducted employing G*Power 3 (Heinrich Heine, University of Dusseldorf, Dusseldorf, Germany) [35]. With 77 patients (42 patients in the KA group and 35 in BCS group) and a type I error (α) of 0.05 by unpaired *t*-test, the study was anticipated to yield a power (1 − β) of 0.80 and 0.93 to detect an effect size q (H1) of 0.65 and 0.80, respectively.

We calculated effect sizes (presented as means ± SD) in the unpaired *t*-test, using Hedges’ g for each parameter, along with the 95% confidence interval (CI) for effect sizes [36].

## Results

### Clinical outcomes

Table 2 presents the postoperative ROM and 2011 KSS for both groups. No significant difference was observed in the mean knee extension and flexion angles between the two groups. One year postoperatively, the KA group showed significantly superior objective knee indicators, patient satisfaction, and functional activity scores. Table 3 summarizes the clinical outcome improvements following modified KA- and guided-motion BCS-TKAs. The scores showed significant improvement in patients who underwent modified KA-TKA compared to those who underwent BCS-TKA, with the exception of the patient expectation score. Neither group had postoperative complications, such as infection, periprosthetic fracture, and nerve and vascular injury. Operative time, intraoperative blood loss, and anesthesia time did not differ significantly between the two groups [KA group vs. BCS group; operative time (minutes)]: KA 93.2 ± 13.5 vs. BCS 94.1 ± 14.6, *P* = 0.8; intraoperative blood loss (mL): KA 46.4 ± 17.7 vs. BCS 44.1 ± 17.8, *P* = 0.59; anesthesia time (minutes): KA 128.1 ± 16.9 vs. BCS 126.1 ± 14.7, *P* = 0.59). Five knees in the KA group and six in the BCS group underwent total intravenous anesthesia, while for all other knees, general anesthesia (inhalation anesthesia) was used. The amount of propofol used in total intravenous anesthesia, as well as the dosage of inhalation anesthesics administered during general anesthesia, did not significantly differ between groups (KA group vs. BCS group; amount of propofol (mg): KA (*n* = 5) 426.0 ± 78.3 vs. BCS 456.7 ± 68.9, *P* = 0.51; amount of inhalation anesthesia: KA 30.2 ± 8.6 vs. BCS 29.8 ± 7.9, *P* = 0.84). The results suggested that the amount of fentanyl, remifentanil, or acetaminophen used for analgesia did not differ significantly between the two groups (KA group vs. BCS group; amount of fentanyl (mg): KA 195.2 ± 63.3 vs. BCS 192.9 ± 60.8, *P* = 0.87; amount of remifentanil (mg): KA 1.33 ± 0.5 vs. BCS 1.4 ± 0.5, *P* = 0.55; amount of acetaminophen (mg): KA 733.3 ± 391.8 vs. BCS 711.4 ± 390.2, *P* = 0.81). For postoperative pain management, a cocktail injection (Anapain 15 mg + Dexate 3.3 mg + Bosmin 0.3 mg + morphine hydrochloride 10 mg + saline 40 mL) was administered into the knee joint at the conclusion of the surgery. Postoperative pain management included celecoxib 200 mg twice daily, along with on-demand administration of either a 50 mg voltaren suppository or oral acetaminophen (400 mg). No significant difference in the frequency of per-request medication usage was observed between the KA group and BCS group (doses per-request medication: KA 4.4 ± 2.4 vs. BCS 4.2 ± 2.0, *P* = 0.62). Regarding the cost of surgical equipment in Japan, for KA-TKA (Zimmer Biomet Inc. Persona CR), the implant price totaled 458,500 YEN, while the accelerometer-based portable navigation system (Zimmer Biomet Inc. iASSIST) cost 90,000 YEN, totaling 548,500 YEN. Meanwhile, for BCS-TKA (Smith and Nephew Inc., JOURNEY II BCS), the implant cost 555,600 YEN in total, and we considered that there was almost no financial difference between the two surgical techniques. In addition, our institution implements the clinical pathway for all TKA surgery cases. Therefore, we considered that there was no significant difference in the average length of hospital stay (KA group vs. BCS group; average length of hospital stay (days): KA 20.3 ± 3.7 vs. BCS 20.5 ± 4.5, *P* = 0.79), as well as in rehabilitation costs and patient medical costs, between the two groups.

**Table 2:** Clinical outcomes in modified kinematically-aligned and guided-motion bicruciate stabilized total knee arthroplasties

	**KA**	**BCS**	***P*-Value**	**Hedges’g (95%CI)**
**Range of motion**				
**Extension, °**	−2.0 ± 3.4	−2.3 ± 3.5	N.S	0.09 (−0.36 to 0.54)
**Flexion, °**	123.5 ± 11.7	125.3 ± 11.9	N.S	−0.15 (−0.6 to 0.30)
**2011 Knee Society Score**				
**Objective knee indicators (100)**	94.3 ± 5.2	90.1 ± 9.6	0.0157	0.55 (0.10 to 1.01)
**Patient satisfaction (40)**	28.4 ± 6.0	23.9 ± 7.1	0.0039	0.68 (0.23 to 1.15)
**Patient expectations (15)**	11.4 ± 2.7	10.2 ± 3.0	N.S	0.41 (−0.03 to 0.87)
**Functional activities (100)**	74.6 ± 14.3	62.8 ± 16.6	0.0013	0.76 (0.30 to 1.23)

The data are given as mean ± standard deviation

*KA* Kinematically-aligned, *BCS* Bicruciate stabilized

**Table 3:** Clinical outcome improvements in modified kinematically-aligned and guided motion bicruciate stabilized total knee arthroplasties

	**KA**	**BCS**	***P*-value**	**Hedges’g (95%CI)**
**Range of motion**				
**Extension, °**	10.6 ± 6.4	8.1 ± 5.6	N.S	0.41 (−0.04 to 0.87)
**Flexion, °**	7.1 ± 9.4	3.0 ± 13.0	N.S	0.36 (−0.09 to 0.82)
**2011 Knee Society Score**				
**Objective knee indicators (100)**	36.5 ± 16.3	27.9 ± 19.3	0.0384	0.48 (0.03 to 0.94)
**Patient satisfaction (40)**	14.5 ± 9.1	8.6 ± 8.8	0.0056	0.65 (0.20 to 1.12)
**Patient expectations (15)**	−0.8 ± 2.8	−1.4 ± 3.2	N.S	0.40 (−0.05 to 0.85)
**Functional activities (100)**	32.8 ± 17.8	20.4 ± 14.5	0.0016	0.75 (0.28 to 1.22)

The data are given as mean ± standard deviation

*KA* Kinematically-aligned, *BCS* Bicruciate stabilized

### Component alignment outcomes

Table 4 summarizes the component alignment in both groups. There was no significant difference in the postoperative HKA in both groups. Differences in postoperative cFCA and cTCA between the groups were significant, and were attributed to different femoral components and insert shapes. Regarding component rotational alignment, the femoral component rotation differed significantly between the groups; however, the tibial component rotation did not. The rotational mismatch also did not show a significant difference between the groups.

**Table 4:** Component alignment in modified kinematically-aligned and guided motion bicruciate stabilized total knee arthroplasties

	**KA**	**BCS**	***P* -value**
**Coronal Alignment**			
**HKA, °**	1.2 ± 1.6 varus	1.0 ± 2.4 varus	N.S
**cFCA, °**	1.5 ± 1.6 valgus	1.0 ± 2.0 varus	*P* < 0.001
**cTCA, °**	2.7 ± 1.0 varus	0.03 ± 1.5 varus	*P* < 0.001
**Rotational Alignment**			
**Femoral Component, °**	−2.6 ± 1.6	−0.6 ± 2.0	*P* < 0.001
**Tibial Component, °**	0.2 ± 1.9	0.4 ± 2.9	N.S
**Rotational mismatch, °**	3.1 ± 1.8	3.0 ± 2.2	N.S

The data are given as mean ± standard deviation

*HKA* Hip-knee-ankle angle, *cFCA* Coronal femoral component angle, *cTCA* Coronal tibial component angle, *KA* Kinematically-aligned, *BCS* Bicruciate stabilized

### Correlation between rotational mismatch and 2011 KSS

**Fig. 3:**
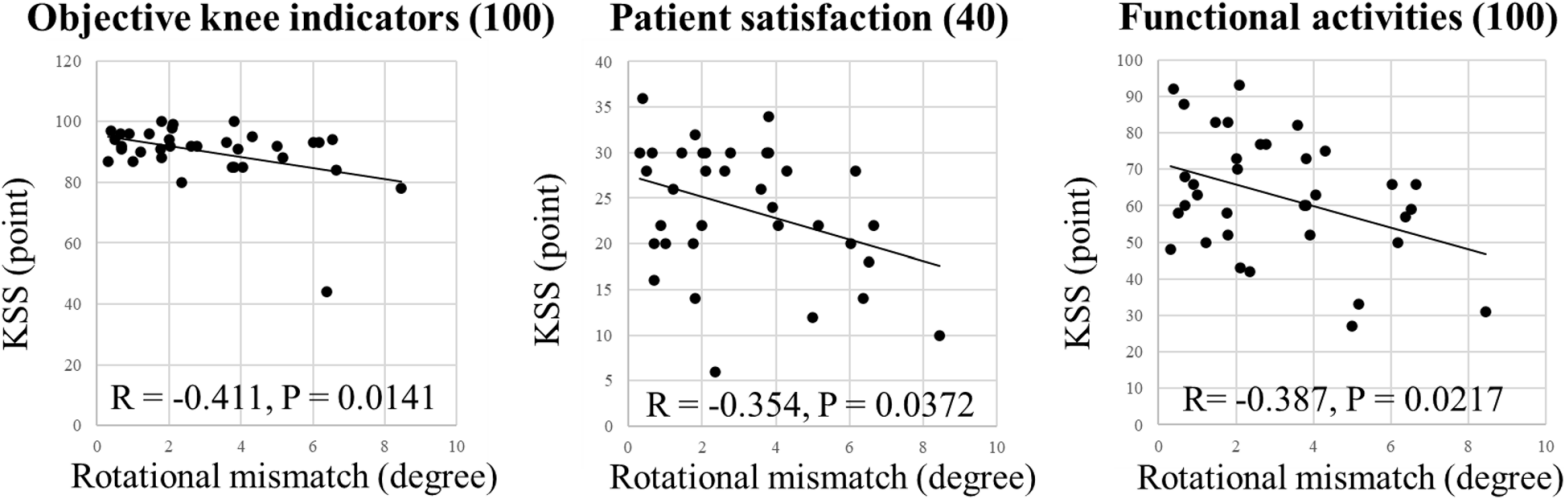
The correlation between the rotational mismatch and the 2011 KSS in guided-motion bicruciate stabilized TKAs. A significant negative correlation was observed between the rotation mismatch and the objective indicators (*R* =  −0.411; 95% CI: −0.655, −0.09; *P* = 0.0141), patient satisfaction (*R* =  −0.354, 95% CI: −0.614, −0.023; *P* = 0.0372), and functional activity (*R* =  −0.387, 95% CI: −0.638, −0.062; *P* = 0.0217) scores (*P* < 0.05 means statistical significance)

Table 5 illustrates the correlations between the component rotational mismatch and the 2011 KSS in each group. No correlations were found between the rotational mismatch and the 2011 KSS in the KA group. Conversely, in the BCS group, significantly negative correlations were observed between the rotational mismatch and the objective indicators, patient satisfaction, and functional activity scores (Fig. 3).

**Table 5:** Correlation between the rotational mismatch and the 2011 Knee Society Score in modified kinematically-aligned and guided-motion bicruciate stabilized total knee arthroplasties

	**KA**			**BCS**		
	***R***	**95%CI**	***P*-value**	***R***	**95%CI**	***P*-value**
**Objective knee indicators (100)**	−0.056	−0.354, 0.252	N.S	−0.411	−0.655, −0.09	0.0141
**Patient satisfaction (40)**	−0.19	−0.467, 0.12	N.S	−0.354	−0.614, −0.023	0.0372
**Patient expectations (15)**	−0.053	−0.351, 0.255	N.S	−0.138	−0.45, 0.205	N.S
**Functional activities (100)**	−0.194	−0.47, 0.117	N.S	−0.387	−0.638, −0.062	0.0217

The data are given as mean ± standard deviation

*CI* Confidence interval, *KA* Kinematically-aligned, *BCS* Bi-cruciate stabilized

## Discussion

One important finding in this study was a significant improvement in the 2011 KSS objective knee indicators, patient satisfaction, and functional activity scores in the KA group compared to the BCS group 1 year after operation. The KA-TKA and BCS-TKA are similarly based on the concept of reproducing physiological knee kinematics; however, their approaches are different. KA-TKA reproduces the patient’s natural knee kinematics through joint resurfacing and preserving the patient-specific soft tissue balance, while BCS-TKA produces generally physiological knee kinematics using guided-motion based on the shape of the implant and insert. Some meta-analyses comparing KA- and MA-TKAs observed that the KSS results supported KA-TKA over a short follow-up period [37, 38]. Additionally, in the most recent meta-analysis, KA-TKA was found to improve several functional outcomes compared to MA-TKA. Nonetheless, more standardized, randomized, and larger studies are needed to improve the quality of evidence [39]. A systematic review of restricted KA-TKA also reported better clinical outcomes and fewer infantile failures than MA-TKA in the short and middle term [40, 41]. In contrast, short-to-medium-term BCS-TKA outcomes have been reported to be equivalent to those of posterior stabilized MA-TKA [22, 23]. Specifically, as shown in this study, the clinical outcome of KA-TKA may be superior to that of BCS-TKA. This is the first report to compare the postoperative clinical results of KA-TKA and BCS-TKA. However, future investigations are warranted to carefully assess long-term clinical outcomes, particularly implant survival.

This study found no difference in component rotational mismatch between the two groups. In the KA group, no correlation existed between rotational mismatch and 2011 KSS scores. However, the BCS group revealed significant negative correlations between rotational mismatch and 2011 KSS subscales, including objective indicators, patient satisfaction, and functional activity. The method of determining component rotation in TKA is an important factor [26, 27]. With respect to the accuracy of tibial component placement using anatomical landmarks as indicators, postoperative rotational component alignment was reported to vary by > 10°. This discrepancy may arise due to the potential obscuration of anatomical landmarks used to determine tibial rotational alignment [42, 43]. Even when the ROM technique was used, reported tibial rotation errors ranged from 27 degrees of external rotation to 12 degrees of internal rotation. These variations can be attributed to factors such as the femoral component, extensor mechanism, ligament balance, and plane of tibial osteotomy [44, 45]. It is important to note that in TKA, errors in the rotational placement of the tibial component may result in a rotational mismatch between components.

Regarding JOURNEY II BCS, Inui et al. observed that intraoperative tight medial flexion gap resulted in reduced intraoperative physiological tibial internal rotation, and that JOURNEY II BCS kinematics may be more susceptible to soft tissue handling than other TKA systems [46]. Kuwashima et al. have reported that the JOURNEY II BCS had a lower contact stress but larger contact area on the anterior tibial post-cam than PS-TKA [47]. In addition, Fujita et al. found negative correlations between femoro-tibial rotational mismatch and clinical outcomes of JOURNEY II BCS TKA [33]. Therefore, it is possible that the restraint for guided-motion is relatively high, and the tolerance for rotational mismatch is low, as indicated by the soft tissue balance and post-cam contact stress analysis. Conversely, with KA-TKA, Nedopil et al. described that internal and external malrotation of the femoro-tibial components within the specified ranges (femur; 3° internal to 2° external, tibia; 11° internal to 12° external) did not affect functional outcomes, as assessed by the Oxford Knee Score and Western Ontario and McMaster Universities Osteoarthritis Index [48]. Therefore, the negative correlation between the rotational mismatch and clinical results observed in the BCS group in this study was expected. In contrast, the postoperative clinical results may have been superior in the KA group, which has a relatively high tolerance for rotational mismatch.

This study had limitations. First, we evaluated only a small population and excluded individuals with valgus and severe varus deformities potentially impacting the results. These exclusions warrant future research. Second, the clinical outcomes were assessed for only 1 year postoperatively. A long-term follow-up should be performed to determine the sustained clinical relevance in the future. Third, intraoperative kinematics was not evaluated. It is necessary to evaluate the intraoperative parameters obtained by the navigation system or tensor in the future. Fourth, KA with tibial-restricted modification was performed using generic instruments in this study, different from the original KA-TKA. Therefore, it could not be accurately compared with previous studies.

## Conclusions

The short-term clinical results following modified KA-TKA in 42 patients showed superior patient satisfaction and functional activity scores, compared with those following BCS-TKA in 35 patients. The BCS group revealed negative correlations between component rotational mismatch and 2011KSS subscales, indicating favorable postoperative clinical outcomes. The KA group exhibited higher tolerance for rotational mismatch, contributing to positive results.

## Data Availability

Data can be obtained by contacting the corresponding author upon request.

## Abbreviations

TKA: Total knee arthroplasty
KA-TKA: Kinematically-aligned total knee arthroplasty
MA-TKA: Mechanically aligned total knee arthroplasty
ROM: Range of motion
BCS: Bicruciate stabilized
ACL: Anterior cruciate ligament
PCL: Posterior cruciate ligament
OA: Osteoarthritis
KSS: Knee Society Score
HKA: Hip-knee-angle
cFCA: Coronal femoral component angle
cTCA: Coronal tibial component angle
SEA: Surgical epicondylar axis
CI: Confidence interval
